# Body language on the pitch: insights into soccer players’ nonverbal behavior at the FIFA men’s World Cup 2022

**DOI:** 10.3389/fpsyg.2025.1699943

**Published:** 2026-01-20

**Authors:** Ingrid Lian, Siv Gjesdal, Yanique Fletcher, Geir Jordet

**Affiliations:** 1 Department of Sport and Social Science, Norwegian School of Sport Sciences, Oslo, Norway; 2 BI Norwegian Business School, Oslo, Norway

**Keywords:** body language, elite sports, football, performance analysis, world cup

## Abstract

Communication is an important aspect in team sports, yet there is a lack of studies looking at intrateam communication during performance. The aim of this study was therefore to explore soccer players’ use of nonverbal behavior (NVB) at the FIFA men’s World Cup 2022. A total of 18,031 distinct NVBs were registered for 143 individual players from 33 games, representing all 32 participating national teams. NVB was divided into tactical and emotional categories. Several statistical tests were performed to investigate the relationship between NVB and potential constraints (e.g., game stage, game halves, roles in a team, geographical location of the national team). The results showed that players displayed more tactical than emotional NVB, and more negative than positive emotional NVB. Moreover, there were differences in NVB expression based on positional roles, but not between captains and other players. NVB expressions changed throughout a game but did not differ between group stage games and knockout games. There were no differences in NVB expressions based on the end result of a game. The findings provide the first empirical examination of quantified NVB of soccer players from the World Cup context. The methodology and findings can be useful for soccer managers, psychologists, researchers and analysts to include psychological measures into game analyses.

## 1 Introduction

Communication is an important factor for team functioning and success in sport (Sullivan et al., 2014; Ishak, 2017). However, most literature regarding communication in sport is concerned with contexts outside the actual competition situation, such as general coach-athlete communication (Shannon et al., 2019). For example, while communication has been linked with coordination, cohesion, commitment, and reaching team goals, to name a few, we lack knowledge regarding intrateam communication during competition (Ishak, 2017). In the present study, this is explored by investigating nonverbal communication during games at the elite level within soccer.

Watzlawick et al. (1967/2014) put forward a theory on human communication, which presents as a useful framework for investigating communication in the sport context (Akpetou and Furley, 2023). The theory outlines five axioms describing the communication process (Watzlawick et al., 1967/2014). Particularly relevant to the current study are axioms 1, 2, and 4. The first axiom states that all behavior has message value, exemplified by the statement that “one cannot not communicate” (Watzlawick et al., 1967/2014, p. 30). This means that it is possible to make inferences about all behavior (Bavelas, 1992), essentially allowing for assessments of behavior through observations. The second axiom presents two levels of communication—content level and relationship level. The former pertains to the message itself and the latter to how the message is being communicated and interpreted (Watzlawick et al., 1967/2014). The relationship level can be revealed nonverbally, for instance by smiling or yelling (Watzlawick et al., 1967/2014). According to Watzlawick et al. (1967/2014), the context (i.e., the environment) is necessary for understanding these two levels of communication. Applied to the current study, which is centered on *in situ* soccer, the context of a game will therefore impact what is communicated and how things are communicated.

The fourth axiom highlights the importance of both verbal and nonverbal modes of communication (Watzlawick et al., 1967/2014). Most human communication happens nonverbally, with estimates ranging between 65 and 95% (Matsumoto et al., 2013). The nonverbal mode is particularly relevant when investigating communication in elite soccer for several reasons. First, players can oftentimes not hear what their teammates are saying verbally, due to both the distance between teammates and the crowd noise. Therefore, focusing on players’ nonverbal behavior (NVB) appears important for investigating in-game communication at the elite level.

When reviewing research on NVB in sport, two types of NVB content become apparent—a tactical aspect and an emotional aspect (e.g., Kraus et al., 2010; Durdubas et al., 2021; Lian et al., 2025). NVBs related to the tactical aspect may work as a way of coordinating a team by expressing intentions (Eccles and Tenenbaum, 2004), for example by pointing to a teammate on where to play the ball. When investigating emotional NVB in sport, one should always include positive and negative components (Lazarus, 2000). Examples of emotional NVB can be throwing one’s arms in the air after a missed opportunity as a sign of anger and frustration, or clapping as a sign of energization and support to the team (Durdubas et al., 2021; Lian et al., 2025). In social groups, emotional expressions could also be used with an intention of apologizing, which is typically associated with feelings of guilt and the desire to correct one’s own behavior in the future (Johnson and Connelly, 2014). Although an apology can be experienced as negative, it is usually perceived as pro-social by others (Bastin et al., 2016). Whereas tactical NVB is considered a way of intentionally communicating, emotional NVB may be both intentional and unintentional (Lazarus, 1999). For example, emotional NVB could be automatic reactions to events (unintentional), or used with a goal to influence someone else (Lazarus, 1999). Looking at an in-game soccer context, Lian et al. (2025) reported that elite soccer players displayed significantly more tactical NVB compared to emotional NVB, but no differences were found between negative and positive NVBs.

NVB during sport competition has been associated with performance in numerous ways, such as frequency of NVB (for a review, see Furley, 2021). For example, several studies have found that higher frequencies of tactical and emotional NVB is associated with better performance (e.g., Durdubas et al., 2021). However, this relationship seems complex, as Moesch et al. (2018) discovered that handball teams displaying a high frequency of NVB during successful play and a low frequency of NVB during unsuccessful play demonstrated improved subsequent performance. In contrast, when teams displayed a high NVB frequency during unsuccessful play and a low NVB frequency during successful play, they tended to perform worse (Moesch et al., 2018). In elite soccer, McLean et al. (2019) asked players to report on a scale from 0 to 4, the amount and benefit of communication they perceived having with teammates during games from 22 matches. The findings suggest that players experienced more intra-team communication (both verbal and nonverbal) in games won and drawn compared to lost (McLean et al., 2019).

Several other factors have also been associated with variations in tactical and emotional NVB in sport. For instance, it appears that game importance is related to NVB. Specifically, it has been found that handball players use more emotional NVB (e.g., celebratory behaviors and touch) in playoff matches compared to league matches (Moesch et al., 2015). This could indicate that athletes’ NVB may differ based on game type (e.g., group versus knockout). Additionally, Leitner and Richlan (2021), found that that an Austrian elite soccer team was involved in 19.5% more emotional situations (including displays of emotional NVB) when fans and spectators were present at the games compared to COVID-19 games, where they were not. There are also indications that NVB expression could change throughout a game due to fatigue and perceived pressure (Furley and Schweizer, 2020). Particularly, it has been argued that as a person gets fatigued and/or stressed, more automatic NVBs and less intentional NVBs are likely to occur (Furley and Schweizer, 2020). This could indicate that the impact of stress and fatigue may differ for emotional and tactical NVBs, as tactical NVB is more intentional, while emotional NVB can be both intentional and automatic.

There could also be associations between NVB and roles within a team, in that specific roles constrain NVB. Cotterill and Fransen (2016) noted that there are certain behavioral attributes (e.g., task-related and motivational-related) associated with being a leader, but to our knowledge, no studies have investigated differences in NVB between captains and non-captains. Moreover, positional roles within a team may also be related to an athlete’s NVB expressions. In soccer, studies show that there are differences in behavioral demands (e.g., clearances, dribbling, passes) based on playing positions (Taylor et al., 2004). Such differences could also manifest in how players use NVB during games. In other words, these findings may indicate that different roles and positions work as constraints shaping athletes’ NVB content.

Another factor which might influence an individual’s NVB displays is nationality, for example whether they hail from individualistic or collectivistic countries. Matsumoto et al. (2009) found that elite judo athletes from countries that are more individualistic with higher population density expressed their emotions more compared to those from collectivistic and lower density population countries. Research has also looked at continent of origin, reporting that some celebrational acts are used cross-culturally while others are more culturally specific. Specifically, Lev et al. (2020) compared soccer players from Europe, Africa and South America participating in the European Champions League, and found that all players used celebrations like appealing to the audience and hands outstretched, while Africans and South Americans used more religious symbols compared to Europeans. Moreover, Halldorsson (2018) found that the Icelandic national soccer team used more acts of communication (including emotional and tactical NVB) than the Argentinian national team (157 registrations compared to 95), indicating differences between national teams.

However, there can be variations within a continent, and some might investigate other geographical divisions than continent, for example geographical regions (e.g., Latin America rather than South and North America; Shuter, 1976). Matsumoto and Hwang (2013a) noted that Middle Eastern countries and East Asian countries differ in their use of gestures, in that the former encourages large and illustrative gestures, while the latter discourages them. In other words, how one groups people from different countries seems to matter, and considerations of language similarities (Matsumoto and Hwang, 2013b) and religion (Culcasi, 2010) are relevant. Based on these findings, it could be that an athlete’s behavior is shaped by where they come from.

A relevant theoretical perspective for the current study of in-situ game behaviors is ecological psychology (Gibson, 1979/1986). This perspective proposes a direct theory of perception, which posits that humans perceive environmental properties directly (i.e., an open performer-environment system), rather than through mental representations as suggested by traditional cognitive theories (Araújo et al., 2023). This approach aligns with Watzlawick et al. (1967/2014) theory on human communication which views human interaction as an open system, highlighting that human interaction and its environment “fit meaningfully together within the same theoretical framework” (p. 104). In recent years, ecological psychology has been applied to the study of behavior in sports, emphasizing that the environment provides opportunities for action, referred to as affordances (Araújo et al., 2006). These actions are limited by specific features or boundaries within the system, known as constraints (Araújo et al., 2023), which can arise from the individual (e.g., personal characteristics of players), the task (e.g., attacking formation), or the environment (e.g., weather conditions, game score, time remaining in the game, competitiveness).

The importance of constraints has led to a constraints-led approach applied in sport coaching (Renshaw et al., 2019). While the ecological approach has predominantly been employed in motor behavior research within sports, recent scholarship has reiterated its applicability to sport psychology (Araújo et al., 2025) and highlighted its relevance to communication research (Patterson and Quadflieg, 2016; Tison and Poirier, 2021a,b). From an ecological perspective, tactical NVB in sport can be understood as actions aimed at influencing the behavior of other individuals (e.g., teammates) by altering their field of affordances (Silva et al., 2013; Tison and Poirier, 2021b). For instance, a player might use NVBs to instruct teammates on positioning or to request the ball. Emotional NVB can be viewed as influenced by constraints, in addition to acting as a constraint on future behavior (Araújo et al., 2020). For example, the psychological characteristics of a player (i.e., individual constraint) and the match score and time remaining in the game (i.e., environmental constraints) may shape how players react with emotional NVB to events. Alternatively, emotional NVBs can be intentionally displayed to constrain the behavior of others (Gabriel, 2021). Perhaps a player expresses anger toward teammates to signal dissatisfaction with their effort, thereby attempting to modify their future actions. Therefore, the ecological view on emotional NVB fits well with Lazarus (1999) emphasizing that emotions can be both intentional and reactive.

Moreover, many of the factors previously associated with NVB in sport can be understood as constraints within the ecological framework. For example, game type (e.g., group versus knockout) and game phase (e.g., first versus second half) may function as task or environmental constraints, while a players’ geographical origin may represent an individual constraint. Additionally, a player’s positional role or status as a team captain could serve as both individual and task constraints. As noted by Furley and Schweizer (2020), little is known about NVB and its constraining factors happening during sport competition, and there is a need for more knowledge to better understand these.

In sum, few studies have systematically investigated elite soccer players’ NVB expressions from entire match performances, with some exceptions, such as Leitner and Richlan (2021), Halldorsson (2018), and Lian et al. (2025). However, each of them has their limitations, such as, including several NVBs in one ‘emotional situation’ (Leitner and Richlan, 2021), investigating the team as a whole, rather than individual analyses (Halldorsson, 2018; Leitner and Richlan, 2021), not using statistical analyses (Halldorsson, 2018), having a small sample size (Halldorsson, 2018; Lian et al., 2025), and mixing men and women (Lian et al., 2025). Additionally, none of these studies have investigated NVB in relation to performance (e.g., game result), and other relevant constraints. Thus, to contribute to this research field, the aim of the present study was to investigate 2022 World Cup soccer players’ use of NVB and explore the relationship between NVB displays and performance, in addition to other variables (i.e., constraints) such as (1) game stage (group versus knockout), (2) game phase (first versus second half), (3) roles (positional and captain role), and (4) geographical region of the national team.

## 2 Materials and methods

### 2.1 Study design

The present study used a naturalistic systematic observational design. This design allowed us to observe the participants in their natural environment, without interfering with them (Cozby and Bates, 2012), which is useful when investigating NVB in sport (Cooper et al., 2020). Methods which can collect data on behavior in the moment, as unobtrusively as possible is particularly useful in ecological research on sport behavior (Araújo et al., 2020), as they allow researchers to capture naturally occurring expressions within a dynamic environment.

### 2.2 Data and participants

The total number of participants in the current study was 143, representing all 32 national teams participating in the 2022 World Cup for men. The mean age was 27.69 (SD = 4.52), and 19 were captains. Of the 143 players included in the statistical tests, there were a total 214 appearances (120 group stage, 94 knockout) from 33 games. There were 103 players coded in one game, 24 players coded in two games, 5 players coded in three games, 7 players coded in four games, and 4 players coded in five games.

We grouped the participants in countries from geographical regions based on location and language similarities, which we deemed relevant for communication research, leading to the following groupings: Europe, Latin America, Middle East, Africa, Southeast Asia, North America, and Oceania. It was also decided to divide the European teams into smaller geographical regions, as this group was larger than the others. As a result, we divided Europe into Northern, Western, Central and Southern.

### 2.3 Procedures

This study was exempt from collecting informed consent from the players. As noted by Winter and Maughan (2009), elite athletes are often not subject to standard informed consent procedures as the nature of their profession includes having their performance monitored and analyzed. We screen recorded videos from an Israeli broadcaster (KAN), who made tactical view and close-up view from the 2022 World Cup games publicly available on their website. Tactical view showed all outfield players at all times, while close-up view zoomed in on one player for a duration of the game. From each game there were two close-up recordings, one that followed players from the home team, and one that followed players from the away team. The close-up recording usually contained between 3 to 6 different players in each game, switching players about every 15 min. However, sometimes the close-up recording showed players for more or less time. In cases where players were shown for less than 10 min they were excluded from the dataset. When using videos from a broadcasting entity, it is important to note that the selection of players is not unbiased. For instance, the broadcaster often captured the same players in several games, often reflecting their high status and/or the significance they have within their respective teams. The players selected for close-up view were often the same from game to game, suggesting there was a bias to include the most profiled players or those who arguably had greater significance for the team.

Sportscode Hudl was used to combine the close-up and tactical videos to be played simultaneously. Close-up view served as the main source for looking at players’ NVB, whilst tactical view was used to provide contextual information when needed (e.g., whether the NVB was directed to a teammate or the referee). Two teams’ close-up recordings were coded with broadcast instead of tactical view, as tactical view for those games could not be used (due to a technical malfunction). There was also one team for which we only had the second half of a game in close-up view. This was also included, as all NVB information was transformed to frequency of NVB displayed per minute in close-up view. Because sound was omitted from some of the recordings (technical malfunction), it was decided to code all games without sound. Some players were analyzed in multiple games, including both group stage games and knockout games. Moreover, NVB was not coded after goals were scored, nor in breaks lasting longer than 90 s (e.g., due to injury), because the focus of this study was in-game play.

#### 2.3.1 Categorizing NVB

The players’ NVBs were quantified using a coding window categorizing NVB into a tactical and an emotional dimension, with sub-categories in each, as has been done in previous research (Lian et al., 2025). NVB was operationalized as any arm-movement indicating a tactical or emotional expression. Arm-movements are visible from a distance, and have been used in previous research on NVB in sport (Durdubas et al., 2021). Tactical nonverbal behaviors (NVB-T) were characterized as movements that indicated cooperation and coordination, and other game-related issues (e.g., trying to influence the referee’s decision). Emotional nonverbal behaviors (NVB-E) included a positive (NVB-E-P; e.g., encouraging teammates by clapping) and negative component (NVB-E-N; e.g., arms in the air after missed opportunity), in accordance with Lazarus (2000). Additionally, an “apology/guilt” behavior was included as it illustrated a distinct behavior in which the analyzed player apologizes for something happening in the game (e.g., raising one’s hand to a teammate after doing a mistake). Table 1 shows the tactical and emotional NVB categories with examples.

**Table 1:** Categorization of the NVBs with examples.

NVB Type	NVB sub-category	Examples
Tactical NVB	Asking for ball	Waving one’s arms to indicate that one is free to receive the ball. Pointing to a specific place where the player wants to have the ball passed to.
	Directing teammates	Pointing to or waving one’s hand to where a teammate should position themselves (offensively or defensively). Gesturing what a teammate could have done in a previous situation.
	Confirming message	Arms out slightly to the side with palm of hand facing down to the ground. Showing a thumbs up. Both are examples that the player has received their teammates message.
	Influencing referee	Raising one arm to signal that the ball is out of play or indicate that an opponent is offsides. Putting one’s finger up and waving it from side to side to indicate that the opponent should not get a free kick. Raising one’s arms to signal to the referee that the opposing team’s goalie has used too much time for their goal kick.
Emotional NVB	Positive	Clapping after someone misses a shot. Showing a thumbs-up to a teammate for good effort. Patting a teammate on the back. Initiating a high-five. Waving one’s arms, clapping hard, or hitting one fist into the palm of the other hand to indicate that their team needs to put in more effort or maintain their current level of effort.
	Guilt/Apology	Raising one’s hand and arm up after the player has made a mistake, signaling an apology to a teammate
	Negative	Throwing one’s arms up to indicate frustration at not getting the ball. Putting the arms out, palms facing up, showing frustration/ disappoint/anger at the referee’s decision. Pointing angrily towards an opponent after being tackled.

The coding of the NVBs is highly dependent on the context of the game, and should be done in motion, not by still photographs. In cases where a coder is uncertain of a behavior indicating negative NVB or tactical influencing the referee, the tactical influencing the referee triumphs. In cases where a coder is uncertain of a thumbs up being positive emotional NVB or confirming message, positive NVB triumphed.

#### 2.3.2 Reliability testing of coders

Inter-rater reliability tests were performed for the five coders in the study, in which the first author was used as the reference for reliability based on previous experience with coding NVBs from close-up video (see Lian et al., 2025). The coders were the first author and four research assistants from a sport science university, and they all had experience with either playing football, or coaching football or team handball. The training period consisted of several phases. Phase one included explanation of the code window and variables included therein. After this, each coder coded the first half of one specific game. The coding was then compared based on the Cohen’s kappa, and results were used to decide areas for improvement in the next phase. These areas differed for each coder, for example, some needed clarification of when a situation should be coded as several NVBs or just one NVB, and others needed clarification on differentiating between the sub-categories. We set a minimum Cohen’s kappa score of 0.61, which is considered as substantial agreement (Landis and Koch, 1977), in order to consider the training successful. Therefore, the training period varied between 3 to 5 weeks, and the final kappa-scores after the training period for the coders were between 0.66 and 0.83. From a previous study using a similar code window, the first author had an inter-rater reliability of 0.66 using close-up (Lian et al., 2025). For more information about the inter-rater kappa scores of the sub-categories, see Appendix A in the supplementary file.

### 2.4 Data analysis

Following the data collection process, a combined spreadsheet of all the coded games was created. We standardized the NVBs to frequency per minute to control for the variation of minutes each player was shown in close-up view. The total number of players that were coded was 150, but it was decided to remove any players who were coded for less than 10 min in total to increase the likelihood that players displayed their “normal” distribution of NVBs. Seven players were therefore removed from further analyses, leaving a total number of participants at 143. The number of players included in each statistical analysis differed based on the research question. For instance, not all players’ teams made it through the group stage games, so naturally, only players that played both group and knockout games could be included in the analysis investigating game stage. The emotional category of guilt/apology were not included in any of the statistical analyses because it was displayed at a very low frequency (or not at all for some players), which made the variable not distributed normally.

Descriptive analyses were performed to investigate mean, standard deviation and frequency of the different NVB categories. Two linear mixed-effects model were performed to investigate the differences in tactical and emotional NVB and in positive and negative emotional NVB (fixed effects), with random intercepts for players and games to account for some players appearing in multiple games, and different players appeared in the same game. In these analyses, each game per player was included. Therefore, a total of 214 observations (games) for 143 players were included, as some players were coded for multiple games. Two repeated measure analysis of variance (RM ANOVA) were performed to investigate whether there were within-subject differences in NVB frequencies based on game importance (group stage versus knockout) and first half versus second half. The assumption of sphericity was checked using Mauchly’s test, and in cases where this assumption was not met, the Greenhouse–Geisser method was used to correct degrees of freedom.

Four Multilevel analyses of variance (MANOVA) were used to investigate between-subject differences in NVB frequency based on different variables (result in game, captain role, positions, geographical area). Only one game per player was selected due to the assumption of independence of observations. The data was screened for outliers using Mahalanobis’ distance. The number of subjects per group had a 3:1 ratio with the number of dependent variables, meaning in cases of 3 dependent variables, a minimum number of subjects would be 9 (Vincent and Weit, 2012). In cases where assumptions of normality for the dependent variables were not met, or equality of covariance matrices were not met, the Pillai’s criterion was used instead of Wilk’s lambda, as recommended by Tabachnick and Fidell (2007). The Bonferroni correction and Benjamini-Hochberg procedure for false discovery rate (FDR) were used to correct for multiple testing in the *post hoc* tests for the RM ANOVAs and MANOVAs.

Please see the Supplementary file for raincloud distributions of the NVB/min based on the different independent variables.

## 3 Results

All means are reported with the standardized NVB/min, meaning number of NVB displayed per minute in close-up view.

### 3.1 Tactical versus emotional NVB and positive versus negative NVB

A linear mixed-effects model was performed to examine differences in tactical and emotional NVB (fixed effect) with random intercepts for players and games. Results showed significant differences between the NVB types, (*F*(1, 213) = 426.15, *p* < 0.001), in that players displayed more tactical NVB (*M* = 2.03, *SE* = 0.06) than emotional NVB (*M* = 0.73, *SE* = 0.06), with a mean difference = 1.30, *SE* = 0.07, 95% CI [1.18–1.43], *p* < 0.001, after controlling for random intercepts of players and game.

A second linear mixed-effects model was performed to examine differences in positive and negative emotional NVB (fixed effect) with random intercepts for players and games. Results indicated that players differed significantly between positive and negative NVB- E, (*F*(1, 213) = 79.73, *p* < 0.001), in that players displayed more negative NVB-E (*M* = 0.45, *SE* = 0.02) than positive NVB-E (*M* = 0.23, *SE* = 0.02), with a mean difference = 0.22, *SE* = 0.02, 95% CI [0.17, 0.27], *p* < 0.001.

### 3.2 Comparison of NVB based on game halves

A 3 × 2 RM ANOVA was used to investigate differences in NVB types (tactical, positive, and negative) between the first and second half. Players who were shown at least 10 min in close-up view in both halves of a game were included, which resulted in 130 players. After the dataset was checked for normality, one case was spotted as an extreme outlier, and was therefore excluded, leaving 129 observations included in the analysis. Results indicated significant differences in NVB registered in first and second half, (*F*(1, 128) = 5.50, *p* = 0.022, η_p_^2^ = 0.41).

*Post hoc* tests revealed that the number of registered NVBs was significantly higher in first half (*M* = 0.964, *SE* = 0.047) compared to second half (*M* = 0.891, *SE* = 0.042), mean difference = 0.073, 95% CI [0.01–0.14], *p* = 0.022. There were also significant differences between the displays of NVB type (*F*(1.17, 149.6) = 346.15, *p* < 0.001, η_p_^2^ = 0.73). Pairwise comparisons using revealed that players used significantly more NVB-T (*M* = 2.08, *SE* = 0.01) compared to NVB-E-P (*M* = 0.25, *SE* = 0.02) and NVB-E-N (*M* = 0.46, *SE* = 0.03). The mean difference between tactical and positive was 1.83, 95% CI [1.60–2.05], *p* = 0.003, and between tactical and negative was 1.62, 95% CI [1.40–1.87], *p* = 0.001. Players displayed significantly more NVB-E-N compared to NVB-E-P, mean difference 0.021, 95% CI [0.13–0.28], *p* < 0.001. The test also revealed a significant interaction effect between NVB type and game half (*F*(1.51, 193.63) = 18.20, *p* < 0.001, η_p_^2^ = 0.12). Pairwise comparisons reveal that the mean of NVB-T goes significantly down in second half. For NVB-E-N and NVB-E-P there were no significant differences in the means between the halves. See Table 2 for specific information of the significant interaction effect.

**Table 2:** Pairwise comparisons of the interaction effect of NVB type based on game half.

NVB/min	Game half	*M* (*SE*)	95% CI	Mean difference	*p*-value
Tactical	1st	2.22 (0.11)	[2.00–2.44]	0.29	0.003*
	2nd	1.93 (0.10)	[1.74–2.13]		
Positive	1st	0.23 (0.02)	[0.19–0.27]	0.04	0.066
	2nd	0.27 (0.02)	[0.23–0.31]		
Negative	1st	0.44 (0.03)	[0.38–0.51]	0.03	0.428
	2nd	0.47 (0.03)	[0.41–0.54]		

The means display NVB per minute. *p < 0.05.

### 3.3 NVB in group games versus knockout games

A second RM ANOVA was performed to investigate whether group stage versus knockout had an impact on displays of NVB/min. Therefore, only players who were coded in group stage games and knockout games for at least 10 min in each game stage were included. This resulted in 35 players included in the analysis. After the dataset was screened, some extreme outliers were detected, and it was decided to perform a winsorizing method in order to deal with the outliers, meaning that the extreme scores were replaced with 3 standard deviations from the mean (Field, 2018).

The RM ANOVA revealed a significant differences in types of NVBs/min (*F*(1.10, 37.24) = 95.36, *p* < 0.001, η_p_^2^ = 0.73), with similar results as the previous test indicating significantly more NVB-T compared to both NVB-E-P and NVB-E-N, as well as significantly more NVB-E-N compared to NVB-E-P. See Table 3 for mean scores and mean differences. There were no significant differences between the total displays of NVB/min based on game type (*F*(1, 34) = 0.012, *p* = 0.915, η_p_^2^ = 0.00). The initial test revealed significant interaction effects (*F*(1.51, 51.19) = 3.53, *p* = 0.049, η_p_^2^ = 0.09). However, when performing pairwise comparisons using the Bonferroni correction and FDR, the effects were no longer significant. See Table 3 for more information.

**Table 3:** Pairwise comparisons of the interaction effect of NVB type based on game type.

NVB/min	Game type	*M* (*SE*)	95% CI	Mean difference	*p*-value
Tactical	Group	2.09 (0.21)	[1.67–2.51]	0.20	0.321
	Knockout	1.89 (0.19)	[1.51–2.28]		
Positive	Group	0.24 (0.03)	[0.19–0.30]	0.02	0.603
	Knockout	0.26 (0.03)	[0.20–0.33]		
Negative	Group	0.36 (0.05)	[0.27–0.46]	0.16	0.144
	Knockout	0.52 (0.07)	[0.38–0.66]		

The means display NVB per minute.

### 3.4 NVB and performance

To investigate NVB displays based on performance, a MANOVA was performed to check for differences in different NVB/min displays based on the result of the game, being win (W), tie (T), and lose (L). In this analysis, the total NVB frequency was used, meaning that if a player was coded in both first and second half, the total amount of NVB was divided by the total number of minutes shown in close-up for that particular game. After screening the data for outliers, the sample size for this test was 139. Using Pillai’s trace, the MANOVA showed no significant differences in mean of NVB/min for NVB-T, NVB-E-P, or NVB-E-N based on the result of the game, *V* = 0.05, *F* (6, 270) = 1.22, *p* = 0.297, η_p_^2^ = 0.03. See Table 4 for mean scores of frequencies for each NVB type based on result in game, and number of players in each group.

**Table 4:** Mean scores of the NVB frequencies based on results, from the non-significant MANOVA.

NVB/min	Result in game		
	W (*n* = 58)	T (*n* = 24)	L (*n* = 57)
	*M* (*SD*)	*M* (*SD*)	*M* (*SD*)
Tactical	1.97 (0.99)	2.31 (0.93)	2.02 (1.14)
Positive	0.23 (0.19)	0.24 (0.17)	0.19 (0.19)
Negative	0.34 (0.24)	0.47 (0.25)	0.35 (0.25)

NVB/min = frequency of NVB displayed per minute in close-up view. W, win; T, tie; L, lose.

### 3.5 NVB, roles and positions

**Figure 1:**
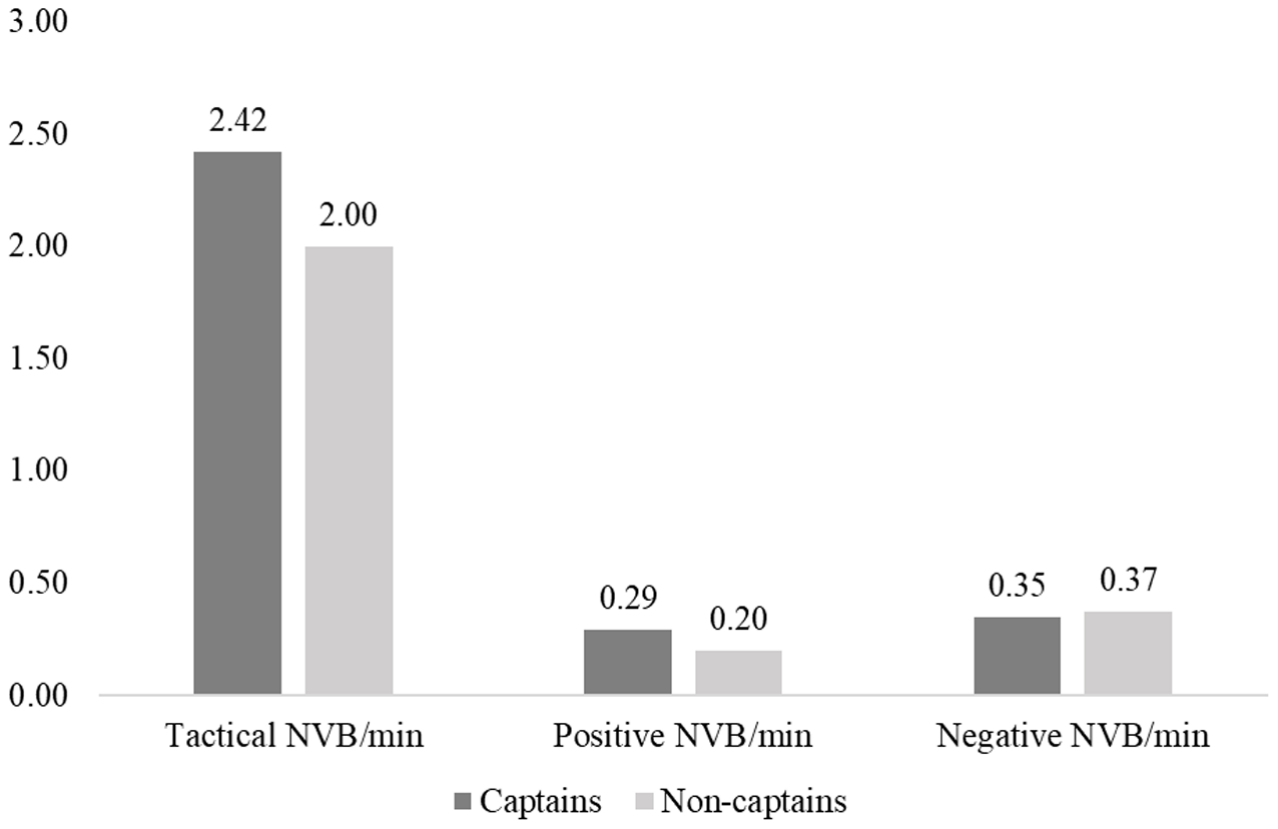
Mean descriptives of tactical, positive and negative NVB between captains (*n* = 19) and non-captains (*n* = 120). The MANOVA revealed no significant differences between the means.

A MANOVA was used to investigate whether there were differences in NVB displays between captains and non-captains. After screening the data for outliers, the sample size for this test was 139 (19 captains, 120 non-captains). The MANOVA showed no significant differences between these means, *V* = 0.043, *F* (3, 135) = 2.01, *p* = 0.115, η_p_^2^ = 0.04. Figure 1 illustrates the means for the captains and non-captains.

**Figure 2:**
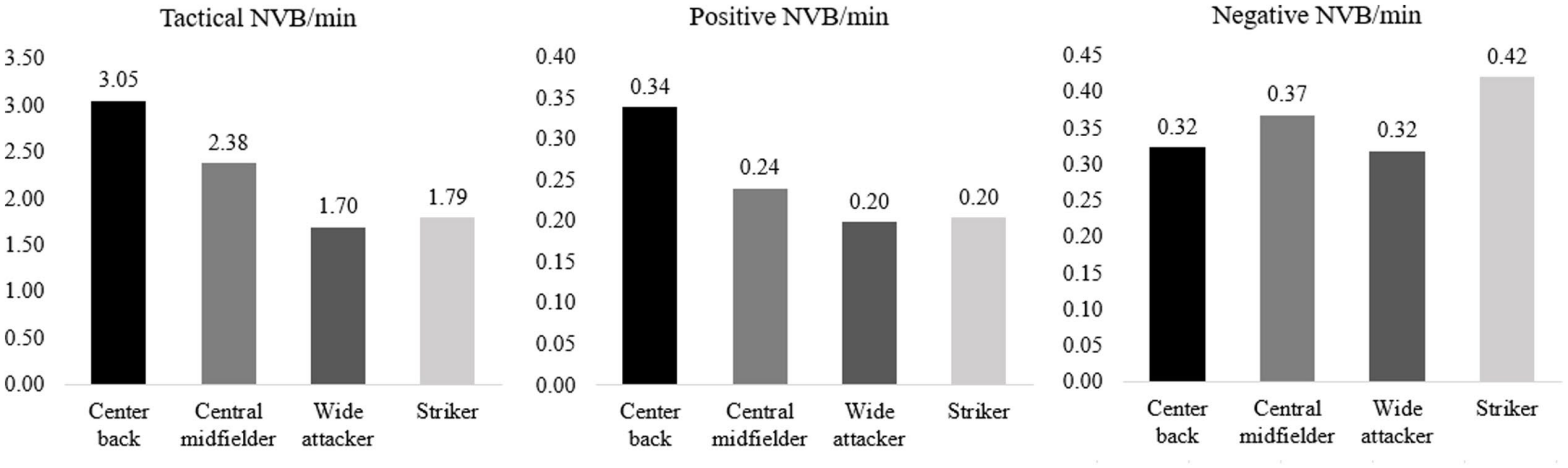
Mean scores of NVB/min (tactical, positive and negative) based on position. For tactical NVB: Significant differences were found between center backs and wide attackers [mean difference = 1.36, 95% CI (0.40–2.31), *p* = 0.028], and between center backs and strikers [mean difference = 1.26, 95% CI (0.31–2.21), *p* = 0.033]. For positive and negative NVB: No significant differences were found between positions.

A MANOVA was conducted to investigate whether there were differences in NVB displays based on positions. After screening the data for outliers, the sample size for the analysis was 130 (11 center backs, 38 central midfielders, 39 wide attackers, 42 strikers). Goalkeepers (*n* = 3) and fullbacks (*n* = 6) were excluded due to insufficient number of subjects in those groups. Using Pillai’s trace, the MANOVA showed that there were significant differences in NVB expression based on position, *V* = 0.28, *F* (9, 378) = 4.24, *p* < 0.001, η_p_^2^ = 0.09. There were significant differences in the use of tactical NVB for some positions, while there were no significant differences in positive and negative NVB based on position. See Figure 2 for information about means and significant differences.

### 3.6 NVB and geographical region

A MANOVA was used to investigate differences in NVB based on the geographical region of the players. After screening data for outliers, there were 6 groups that were compared (Europe = 57, Latin America = 24, Middle East = 21, Africa = 11, Southeast Asia = 11, North America = 9), and one group that was excluded due to few subjects (Oceania = 6). Using Pillai’s trace, the MANOVA showed no significant differences in mean of NVB/min for NVB-T, NVB-E-P and NVB-E-N based on geographical region, *V* = 0.10, *F* (15, 381) = 0.86, *p* = 0.614, η_p_^2^ = 0.03. See Table 5 for mean scores of frequencies for each NVB type based on geographical region.

**Table 5:** Mean scores of the NVB frequencies based on geographical region.

NVB/min	Geographical Region					
	European (*n* = 57)	Latin-America (*n* = 24)	Middle East (*n* = 21)	Africa (*n* = 11)	South East Asia (*n* = 11)	North America (*n* = 9)
	*M* (*SD*)	*M* (*SD*)	*M* (*SD*)	*M* (*SD*)	*M* (*SD*)	*M* (*SD*)
Tactical	2.21 (0.91)	1.74 (1.16)	2.12 (0.74)	2.00 (1.39)	1.81 (0.96)	2.13 (1.44)
Positive	0.25 (0.20)	0.19 (0.16)	0.17 (0.15)	0.18 (0.10)	0.21 (0.22)	0.19 (0.31)
Negative	0.40 (0.26)	0.30 (0.26)	0.45 (0.27)	0.38 (0.24)	0.25 (0.08)	0.35 (0.17)

NVB/min = frequency of NVB displayed per minute.

A MANOVA was used to investigate differences in NVB based on geographical regions within Europe. Four groups were created, being Northern Europe (*n* = 13), Western Europe (*n* = 14), Central Europe (*n* = 12) and Southern Europe (*n* = 19). Using Pillai’s trace, the MANOVA showed no significant differences in mean of NVB/min for NVB-T, NVB-E-P and NVB-E-N based on culture, *V* = 0.13, *F* (9, 162) = 0.78, *p* = 0.632, η_p_^2^ = 0.042. See Table 6 for mean scores of frequencies for each NVB type based on geographical region of Europe.

**Table 6:** Mean scores of the NVB frequencies based on geographical region of Europe.

NVB/min	Geographical region in Europe			
	Northern Europe (*n* = 13)	Western Europe (*n* = 14)	Central Europe (*n* = 12)	Southern Europe (*n* = 19)
	*M* (*SD*)	*M* (*SD*)	*M* (*SD*)	*M* (*SD*)
Tactical	2.36 (0.86)	2.00 (0.58)	2.13 (0.95)	2.33 (1.11)
Positive	0.26 (0.18)	0.18 (0.12)	0.29 (0.26)	0.28 (0.21)
Negative	0.51 (0.24)	0.43 (0.24)	0.30 (0.30)	0.42 (0.36)

NVB/min = frequency of NVB displayed per minute.

## 4 Discussion

The present study is the first to examine soccer players’ use of NVB in a FIFA Men’s World Cup. A total of 143 players were analyzed with a total number 18,031 registrations of NVB. There were several noteworthy results from the study. For example, (1) players used more negative than positive emotional NVB, (2) the number and type of NVBs seemed to change throughout a game, in that tactical NVB was higher in the first half compared to the second half, and the frequencies of NVB did not differ based on (3) game type, (4) end result in the game, (5) players’ geographical region, or (6) captain role. However, (7) there were differences in NVB based on player position.

Regarding Watzlawick et al. (1967/2014) second axiom about content, players used both tactical and emotional NVB as content throughout games, but to a varying degree. In comparison to a previous study using similar methods looking at elite soccer players (see Lian et al., 2025), the current study offers both similar and contrasting findings. Similarly, it was found that players used more tactical NVB compared to emotional NVB. This could suggest that in-game play may afford more tactical NVB compared to emotional NVB. In contrast to the previous study, the present study found significant differences in positive and negative emotional NVB, in that players used more negative NVB. Whereas the current study investigated games from the World Cup, the previous study included games outside a tournament context (e.g., Nation’s League, European qualifier and friendly games). One explanation for the difference in negative and positive NVBs could be that experiences of psychological stress and pressure are higher in a World Cup, which may be linked to more experiences of negative emotions (e.g., anger) for the soccer players (Lazarus, 1999). Additionally, the previous study included both men and women, whereas this study only included men, which could have an influence on the findings.

As theorized by Watzlawick et al. (1967/2014), the context is important in communication, and can impact the content of communication. From an ecological perspective, contextual factors are made up from the interacting individual and environmental constraints (Button et al., 2020). There could be indications of interacting constraints in the present research, for instance regarding game halves. Considering the changes in NVB expressions between game halves, it is worth mentioning other investigations from the 2022 World Cup. It has, for example, been reported that overall running distance and high-intensity running distance decreased in second half compared to first half (Chen et al., 2025). Such declines are often linked to physical fatigue (Dambroz et al., 2022), which could be viewed as an individual constraint in the present study; as fatigue increases, tactical NVB decreases, while no changes were found in positive and negative NVB. Emotional NVB contains automatic expressions, not only intentional (Lazarus, 1999), thus, perhaps tactical NVB requires more deliberate effort than emotional NVB. This supports arguments from Furley and Schweizer (2020), who have stated that fatigue is more likely to constrain intentional NVB compared to automatic NVB.

When it comes to another environmental constraint, game stage, no differences were found related to overall NVB displays, and no interaction effects were detected for the sub-categories of NVB. This contrasts with findings from team handball, which has found differences in emotional NVB between league and playoff matches, including celebratory behaviors (Moesch et al., 2015). In regards to theorizing by Lazarus (1999), who stated that athletes are more likely to have automatic responses and negative emotions in high-stake stressful situations, one could speculate that group and knockout games are perceived equally important and stressful for the players, hence no difference in emotional NVB.

The current study found no significant differences in NVB displays based on the end result of the game. This contrasts to some previous studies, reporting that winning teams displayed more NVB than losing teams (e.g., volleyball; Durdubas et al., 2021), but supports other studies which have not found evidence for such relationship (e.g., tennis; Lausic et al., 2009). This could indicate that there are differences between sports, highlighting the importance of doing sport-specific research on NVB. However, finding links between NVB and soccer performance is probably much more nuanced than only looking at the end result (see, for example, Moesch et al., 2018), hence, other measures should be considered in the future.

Regarding roles and positions on the field, it was found that NVB displays did not differ between captains and non-captains. Positional roles, on the other hand, seemed more relevant, as findings show that center backs displayed more tactical NVB than wide attackers and strikers. These findings could indicate that the positioning on the field constrains the content of NVB displays. For example, being in a more central role and behind on the field may give greater overview and more opportunities to direct teammates. It would be interesting to consider in future research whether it is the roles themselves that work as task constraints on certain behavioral expressions, or whether certain types of personalities work as individual constraints, attracting players to different positions. Nonetheless, it seems reasonable to think that being in different positions on the field affords different amounts of tactical NVB displays during play, similar to other positional differences, like the number of clearances (Taylor et al., 2004). However, playing position appears to not be related to emotional NVB expressions, which could be explained by many emotional NVBs are automatic, hence players seem react to events in similar ways.

The present study did not find any differences in NVB displays based on players’ geographical region. The current findings could indicate that tactical requirements and emotional affordances for players and teams across the world are similar, and this could be related to their similar objectives: teams want to score and avoid the opponents from scoring. Regarding emotional NVB, this study’s results are in contrast to previous findings in sport which have found differences in emotional NVB based on where athletes are from (Matsumoto et al., 2009; Lev et al., 2020). However, the other studies investigated NVB related to goal celebration and NVB shown after competition, which this study did not include. It could therefore indicate that where a player comes from is not a constraining factor on NVB content during play, but rather after goals are scored and the game is finished.

### 4.1 Practical implications

As the existing research on NVB during sport competition is limited (Furley and Schweizer, 2020), this study has potential implications for coaches and practitioners, also outside the World Cup context. For instance, it offers a direct and systematic way of investigating soccer players’ observed behavior from real games. This may also be a starting point for including a psychological measure into game analyses, where soccer analysts can create communicative profiles for their players. These assessments could be used to work on team dynamics and cohesion, as these variables are related to communication (Ishak, 2017). Coaches can establish baseline player and team NVB norms, and changes in NVB at the team or player level can perhaps be an early signal to changes in group dynamics or individual player psychology. Additionally, coaches and staff members could address this study’s findings in team meetings, which could help bring about awareness of players’ own nonverbal communication. Knowing how the best players and national teams in the world communicate nonverbally could provide useful benchmarks and information for teams around the world. It is important to note, however, that these benchmarks are of international men’s standard, and NVB displays may vary based on level and speed of the game, which highlights the necessity for research on professional leagues, in addition to youth and women’s football.

The positional differences might indicate that coaches should have different expectations for their players’ communication on the pitch based on positions, similarly to motor behaviors (Taylor et al., 2004). In line with the constraints-led approach to training (Renshaw et al., 2019), coaches could use the current findings as a way to design practices with an emphasis on team communication. For instance, applying tactical NVB requirements towards the end of training to work on enhancing tactical NVB when players become fatigued. Although we do not address whether tactical, positive, and negative NVB are constructive or destructive in this study, that is something coaches may do themselves within their team.

### 4.2 Limitations

There are some limitations that are important to note and should be taken into consideration when interpreting the findings. One is that players were only shown for segments of the games. Full-game recordings of each player would have allowed for more in-depth analyses of NVB changes throughout a game. Moreover, looking at arm-behaviors might lead to a bias in favor of tactical NVBs, as research on emotion is often concerned with facial expression (Barrett et al., 2019). However, it is difficult to include this in our analyses as players are not always faced toward the camera. As previously mentioned, there might be better ways to investigate the NVB-performance relationship than using the end result of a game, for instance by looking at NVB changes before/after goals scored, or before/after successful ball execution. Additionally, there could be other elements included in the investigation of NVB, for instance the intensity and size of the NVB, which might have revealed differences based on geographical origin of players.

There was also a selection bias in that the players that were filmed in close-up were decided by the TV station, and not the researchers. The players shown in close-up view were usually the most highly profiled players on their respective national teams. To account for this, it will be important in future research to analyze all the players in a team, not only the most profiled ones. In addition, the present study only investigated the content aspect of communication, and not the relational aspect, and how players’ NVB might impact their teammates. Future studies could address this by doing interviews with players or investigating to whom the NVB is directed. Regarding the content, some NVBs were harder to distinguish than others (e.g., tactical influencing the referee versus negative emotional expression), and we therefore had to apply decision rules.

Finally, it is important to note some issues regarding reliability. First, while the overall kappa scores were considered as substantial agreement, three of the coders only had fair inter-rater agreement (0.37–0.38) with the first author on the emotional sub-category. This could be because it might be more difficult to code emotional NVB, but it could also be because fewer subjects were coded in the emotional category, which leads to few errors creating smaller kappa-scores. Also, in the current study we did not perform any intra-rater reliability tests. These issues can be addressed in future research by performing both inter- and intra-rater agreement analyses, and possibly to select more emotional situations that each coder codes.

## 5 Conclusion

The present study offers new information regarding elite soccer players’ use of NVB throughout games and is the first of its kind to do so in a World Cup context. This study offers insights into NVB content and variables that may be considered as constraints on NVB (e.g., game type and positional roles), and could be useful for coaches, practitioners and researchers within soccer. In line with Watzlawick et al. (1967/2014), NVB appears relevant for investigating communication, and tactical and emotional NVB may be considered as central aspects of in-game communication in soccer. Finally, several factors appear to be related to NVB, which from an ecological perspective could be viewed as constraints (Renshaw et al., 2019).

## Data Availability

The datasets presented in this article are not readily available because they contain data from high-profile athletes who could be identified through dataset access. Requests to access the datasets should be directed to the IL, ingridli@nih.no.
