# Supplementary material for: Body language on the pitch: insights into soccer players’ nonverbal behavior at the FIFA men’s World Cup 2022
Source: Front Psychol. 2026 Jan 20;16:1699943. doi: 10.3389/fpsyg.2025.1699943 (PMC12865708; doi:10.3389/fpsyg.2025.1699943)

**Appendix A**

Overview of the inter-rater reliability using Cohen’s kappa, including the sub-categories of tactical and emotional NVB. In addition, an overview of the number of games and players each coder was responsible for.

|  | Coder 1 – Coder 2 (D) | Coder 1 – Coder 3 (M) | Coder 1 – Coder 4 (E) | Coder 1 – Coder 5 (A) |
| --- | --- | --- | --- | --- |
| NVB total (95% CI)  Number of NVB instances | .71 (.59-.84)  68 subjects | .66 (.53-.79)  66 subjects | .66 (.55-.77)  82 subjects | .83 (.73-.92)  82 subjects |
| NVB tactical (95% CI)  Number of NVB instances | .67 (.50-.85)  52 subjects | .58 (.40-.76)  42 subjects | .61 (.47-.74)  64 subjects | .80 (.67-.92)  66 subjects |
| NVB emotional (95% CI)  Number of NVB instances | .37 (.07-.68)  20 subjects | .38 (.07-.68)  26 subjects | .38 (.13-.62)  23 subjects | .75 (.47-1.00)  15 subjects |

| Coder | Games coded | Players coded |
| --- | --- | --- |
| 1 | 5 games | 16 players |
| 2 | 9 games | 34 players |
| 3 | 5 games | 39 players |
| 4 | 9 games | 62 players |
| 5 | 11 games | 74 players |
| *Note.* Some coders coded the same players, but never the same players from the same game. | | |

**Raincloud distributions of NVB/min based on different independent variables**

1. **Distribution of NVB/min based on halves**

**
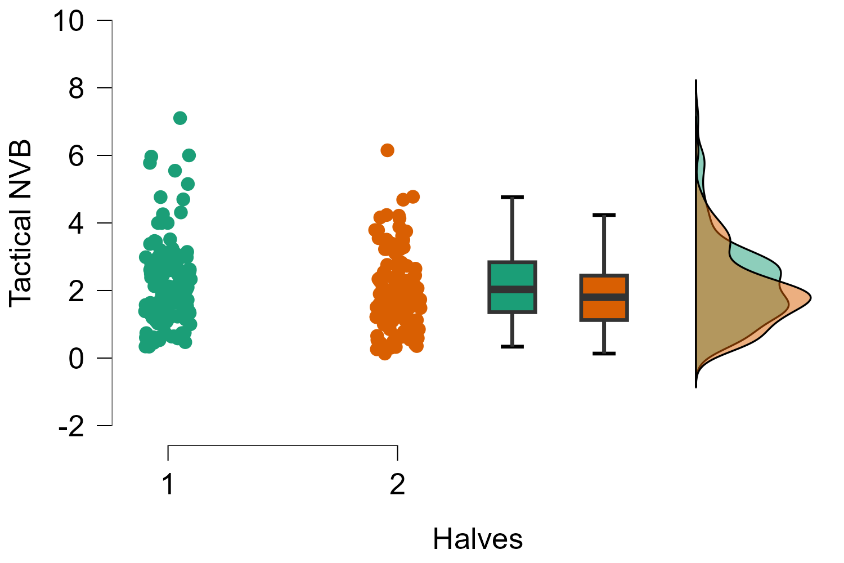

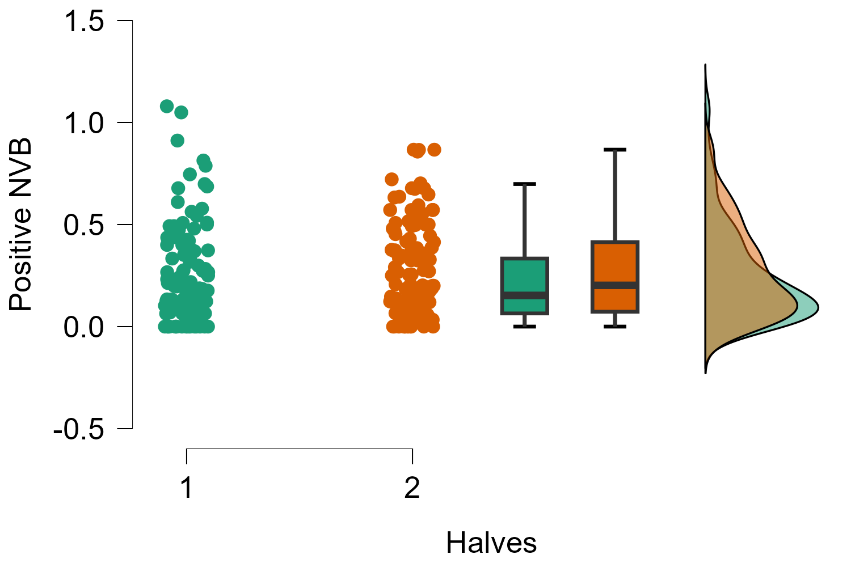
**
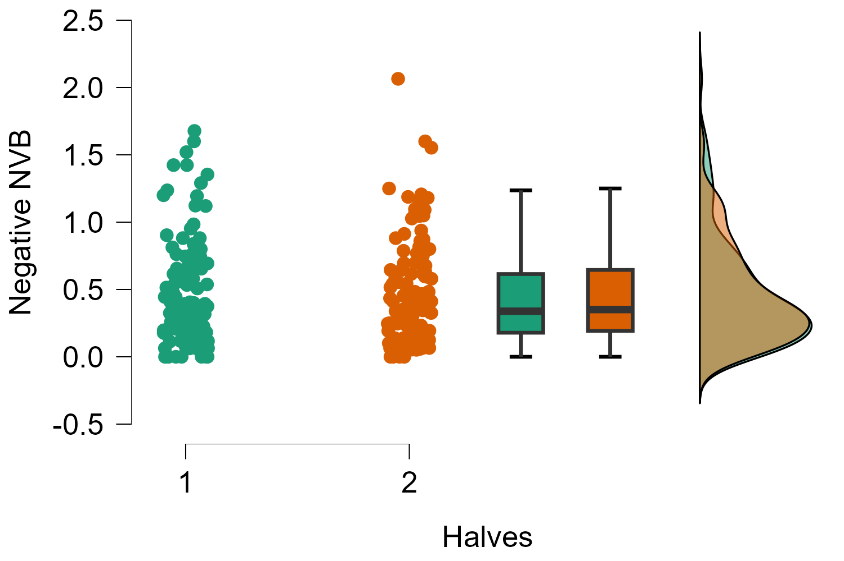


1. **Distribution of NVB/min based on game type**


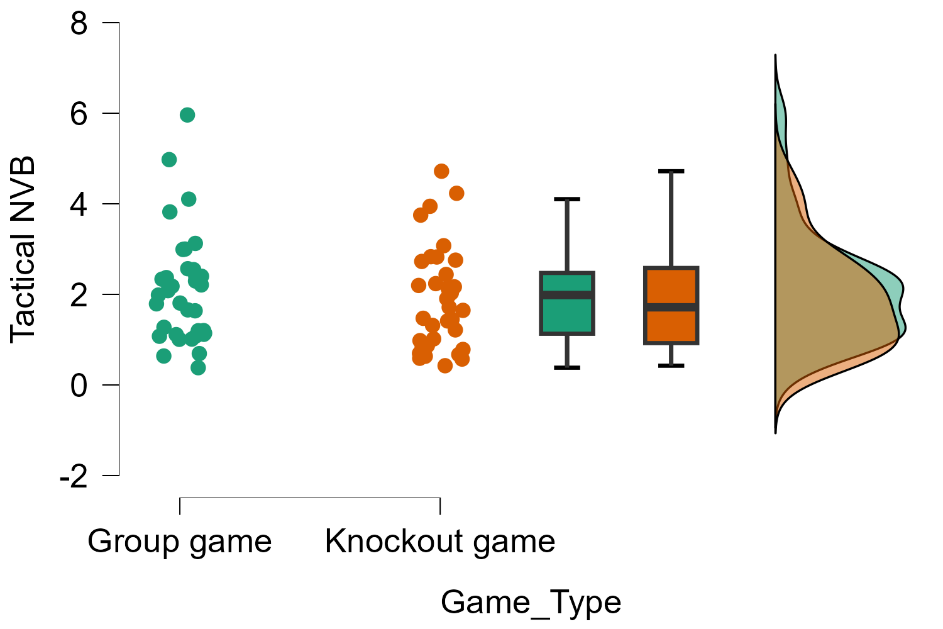

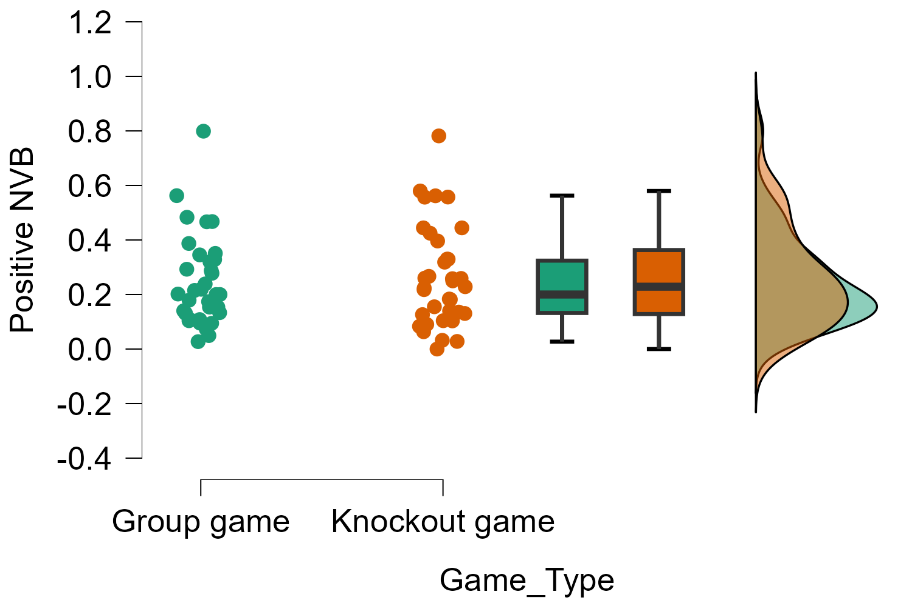


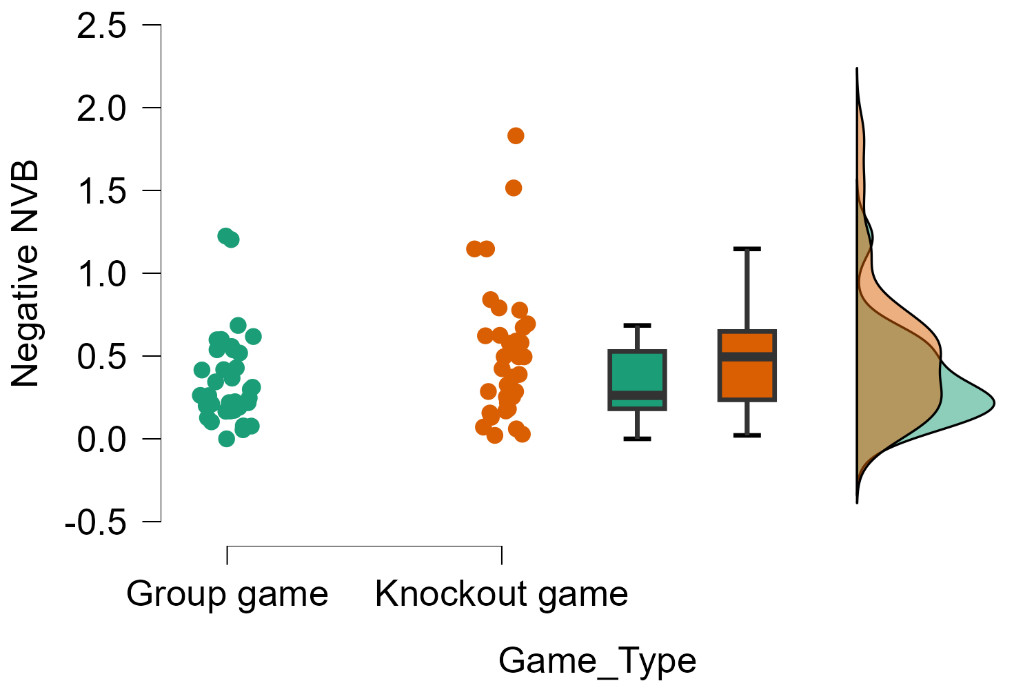


1. **Distribution of NVB/min based on result in game**


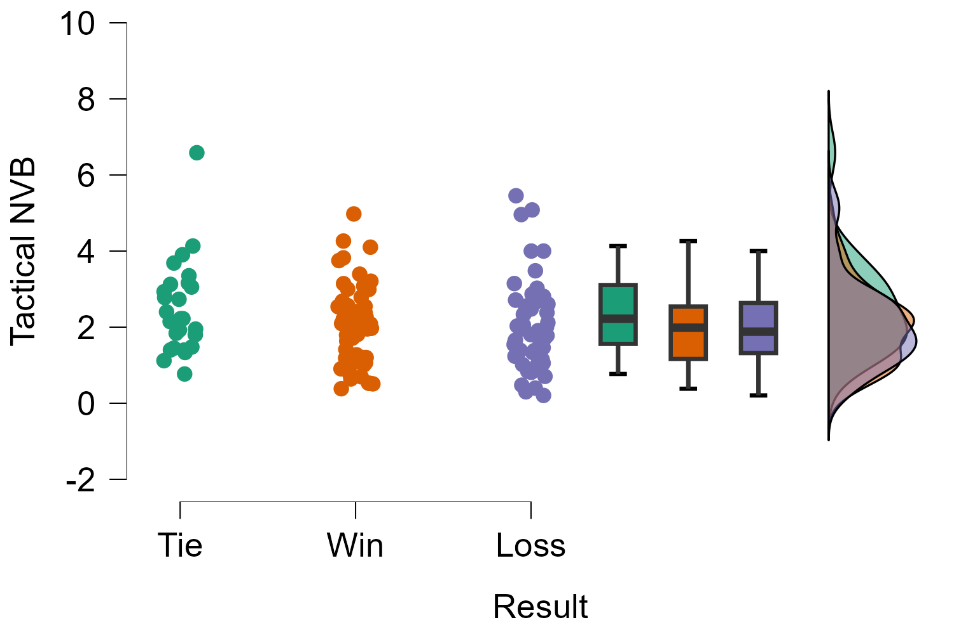

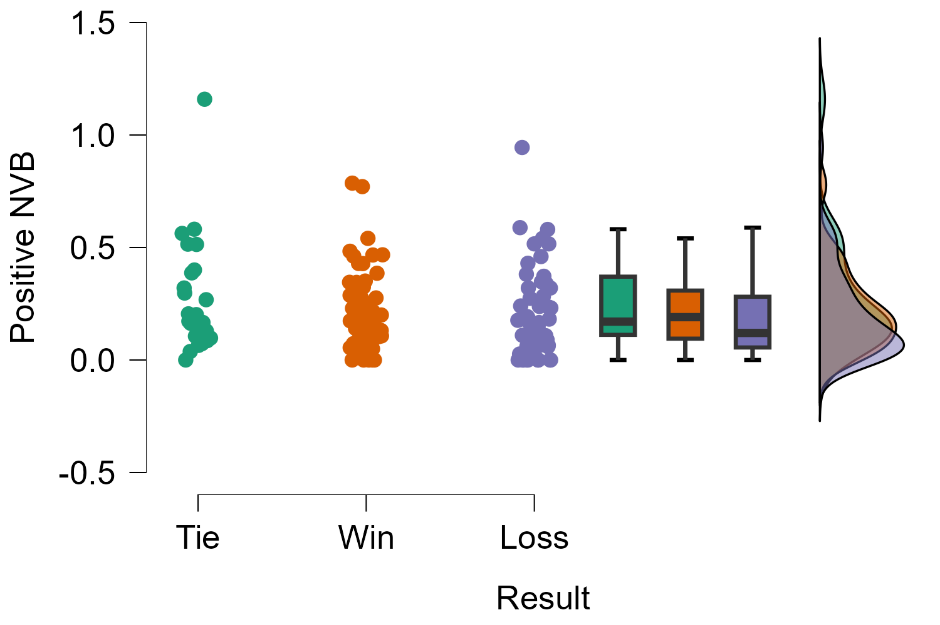


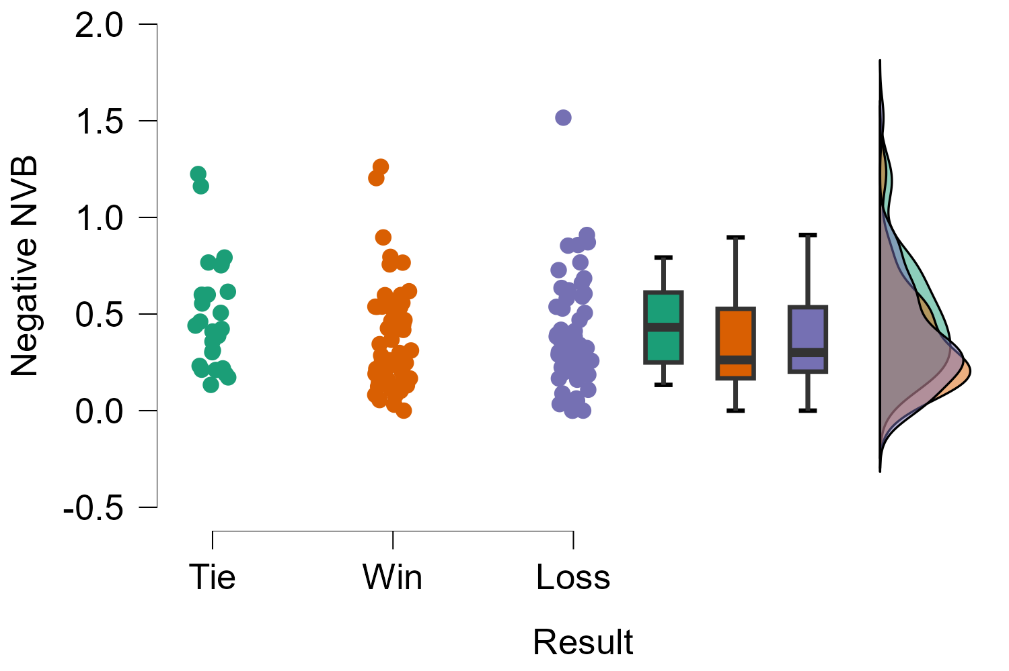


1. **Distribution of NVB/min based on captain role**

**
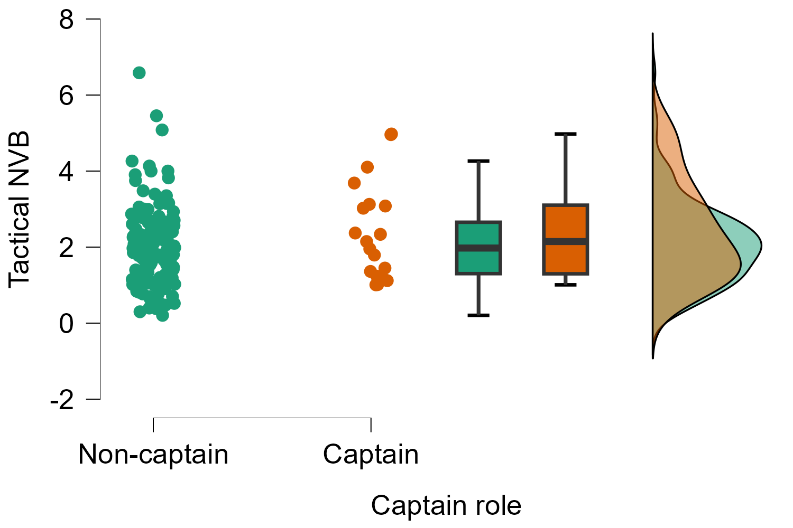

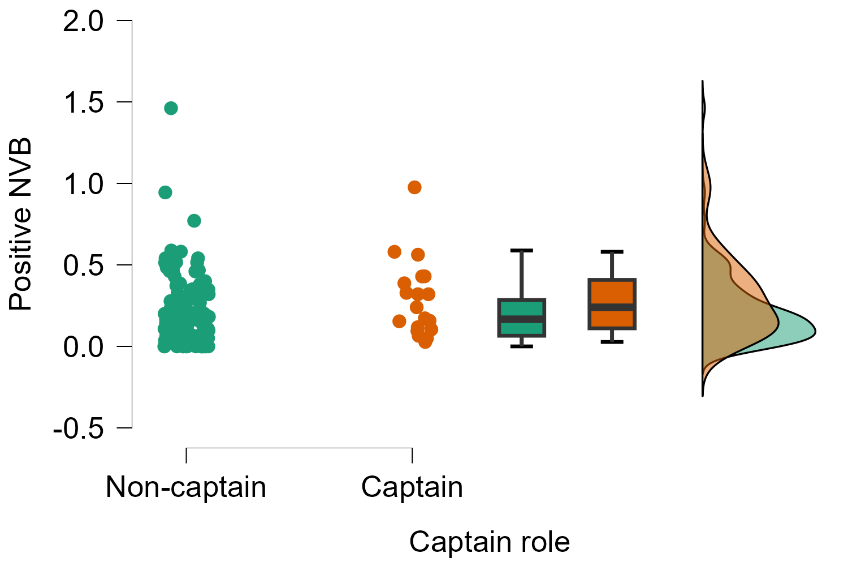
**

**
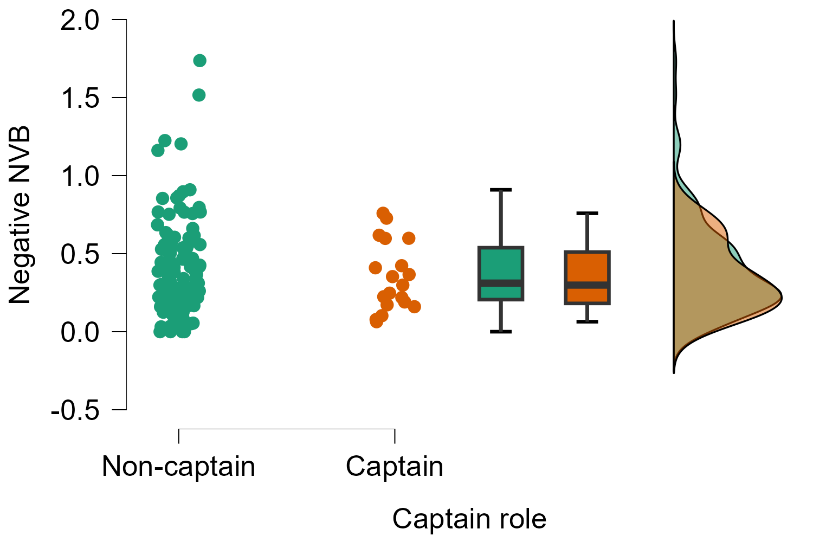
**

1. **Distribution of NVB/min based on position:**CB = Center back, CM = Central midfielder, WA = Wide attacker, S = Striker.

**
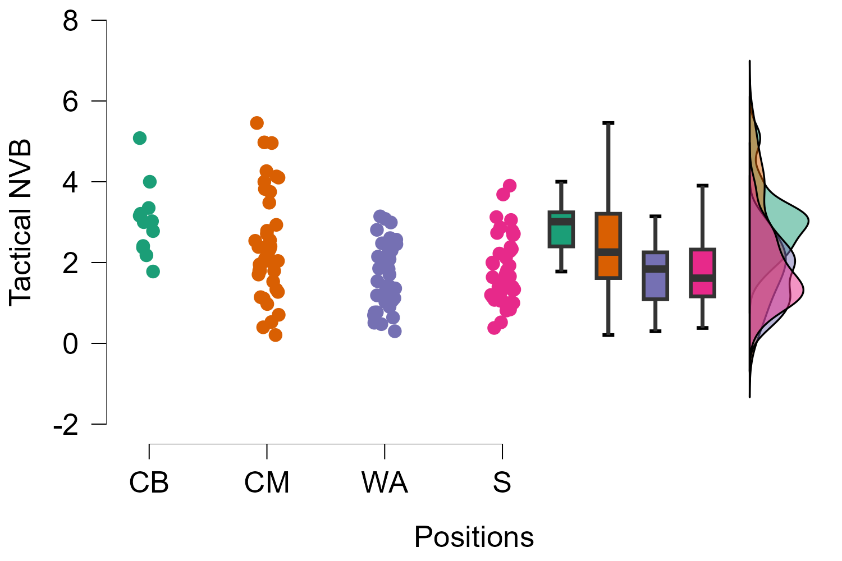

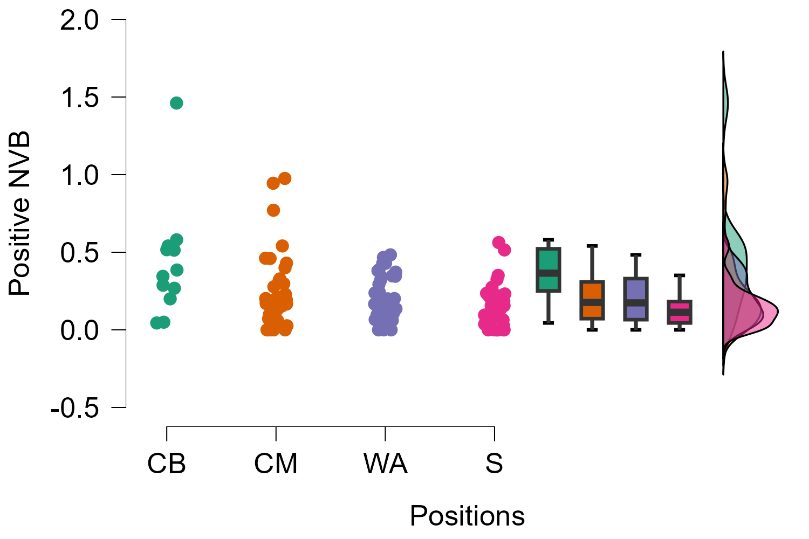
**

**
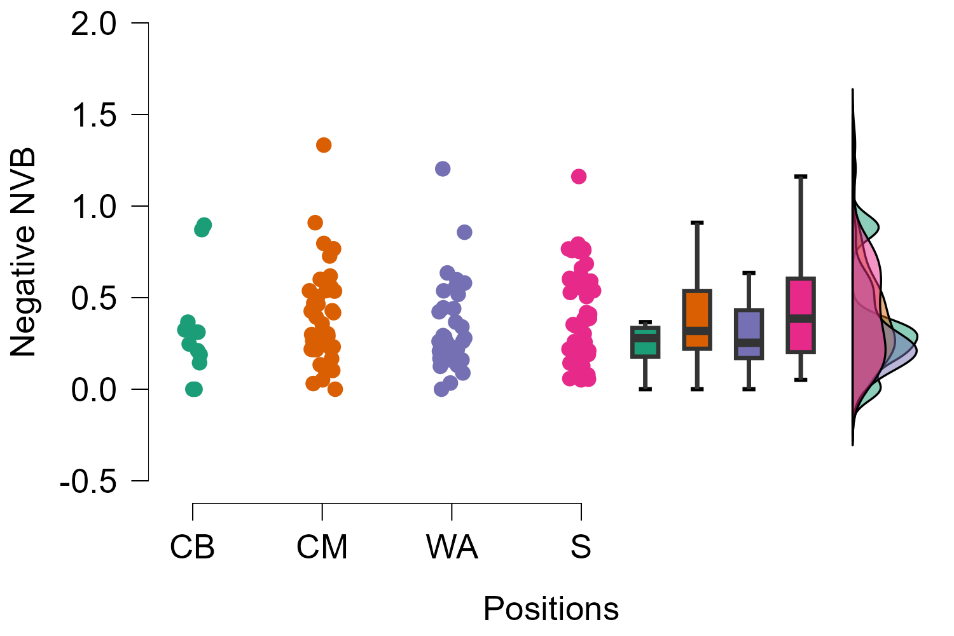
**

1. **Distribution of NVB/min based on geographical origin**ME = Middle East, Eu = Europe, Af = Africa, Oc = Oceania, LA = Latin America, SEA = South East Asia**
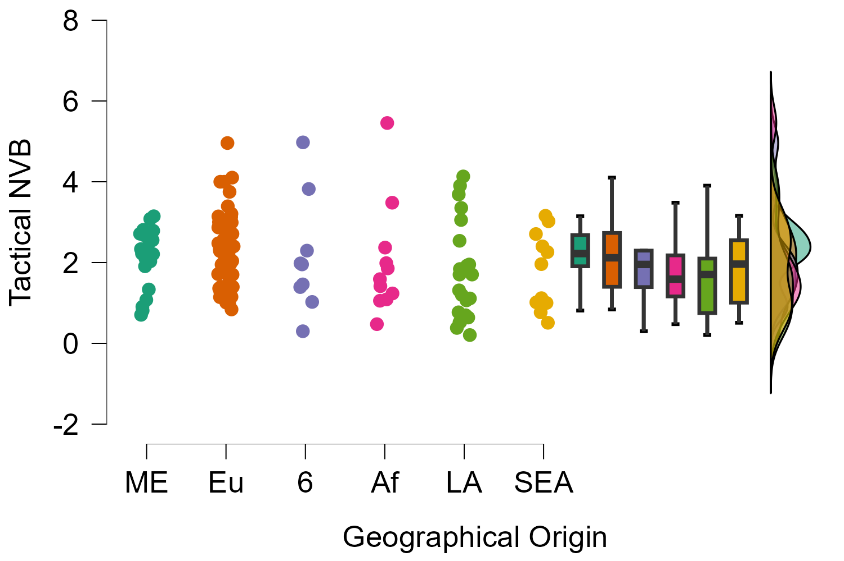
**
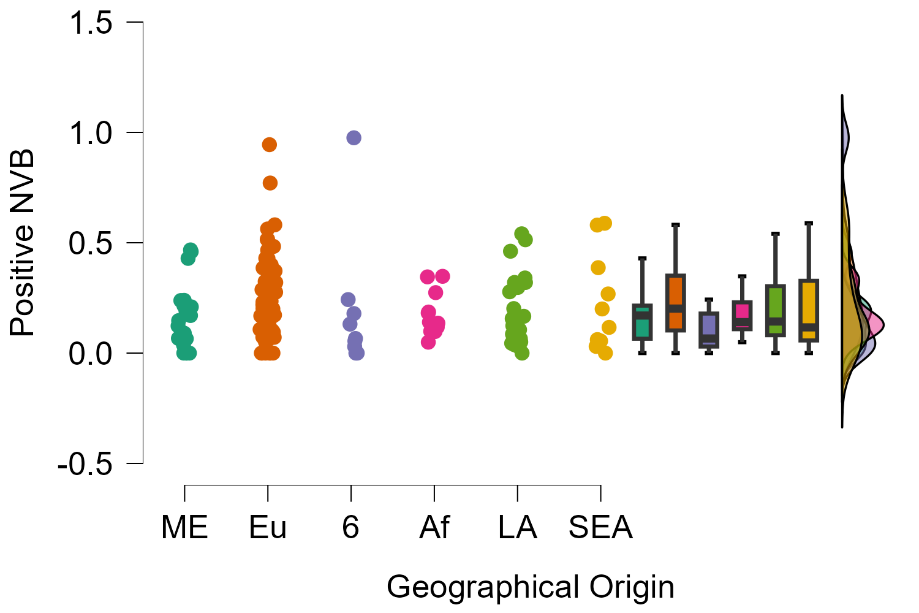


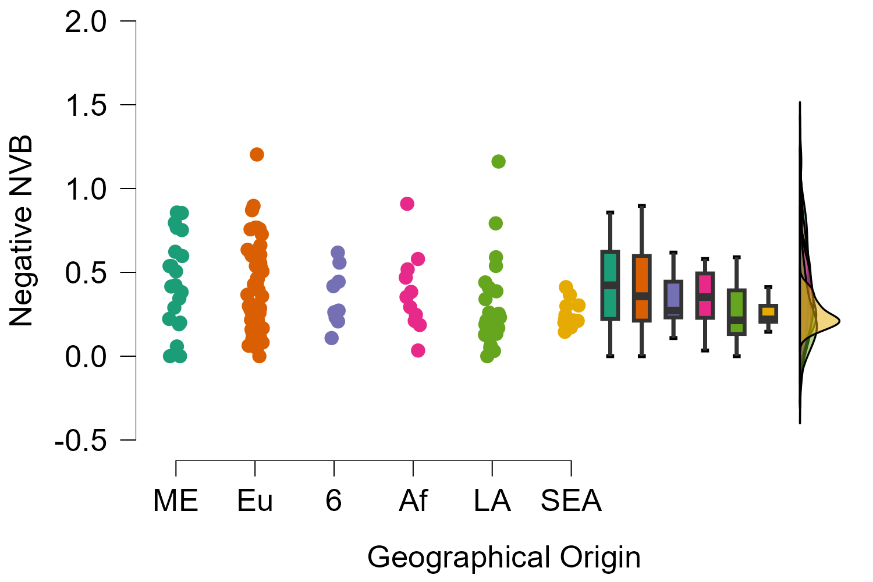


1. **Distribution of NVB/min based on geographical origin (within Europe)**


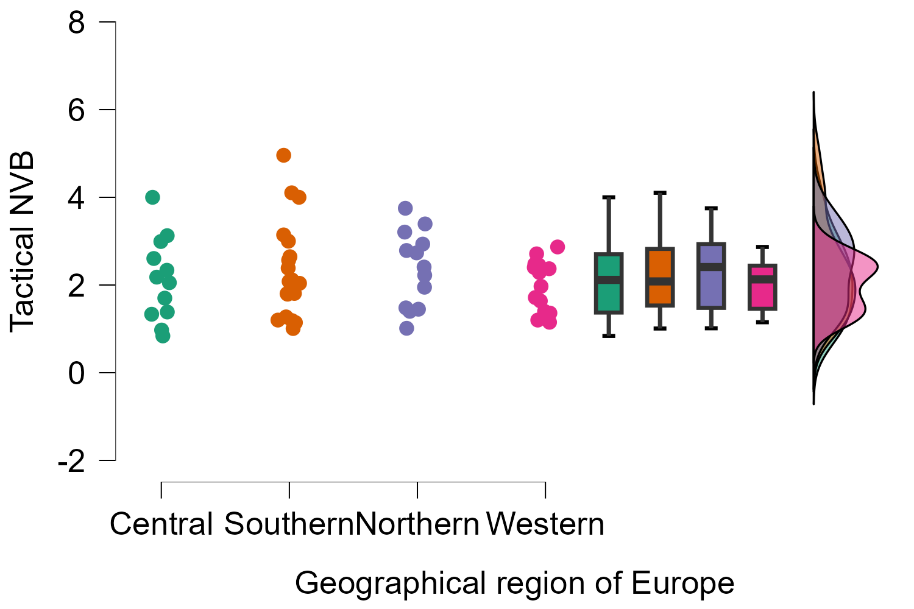

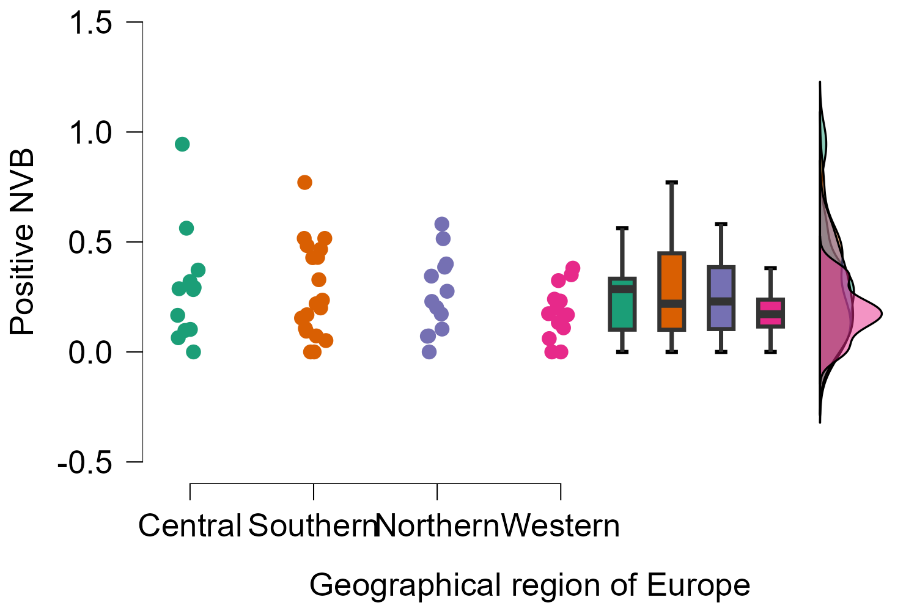

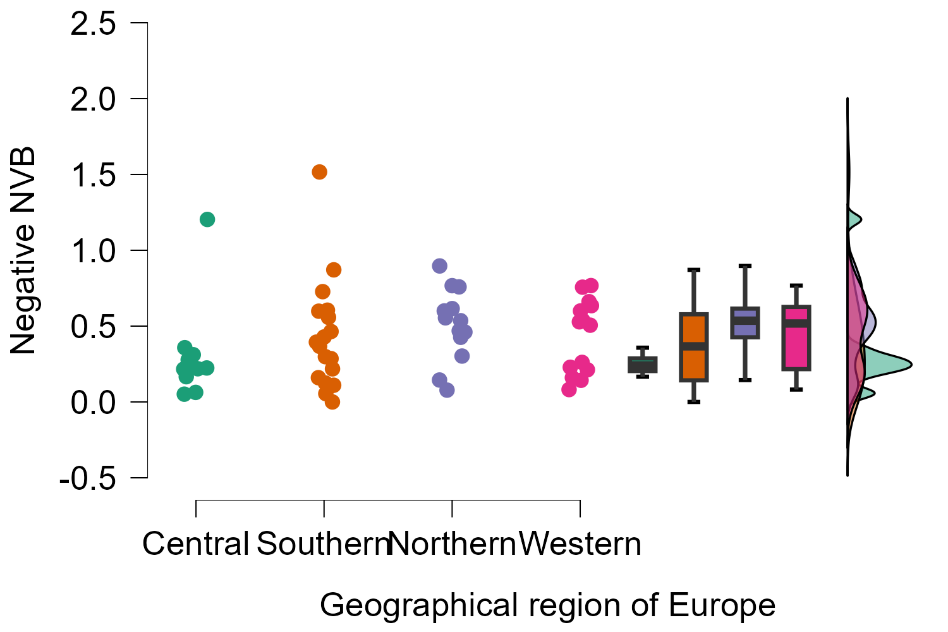

Supplement: Supplementary file 1 [file Data_Sheet_1.docx]
